# Impact of Absence Seizures on Physical Activity Levels in Children: A Cross-Sectional Study

**DOI:** 10.3390/children12060791

**Published:** 2025-06-17

**Authors:** Martina Gnazzo, Valentina Baldini, Marco Carotenuto, Giulia Pisanò, Giovanni Messina, Fiorenzo Moscatelli, Maria Ruberto

**Affiliations:** 1 Department of Biomedical, Metabolic and Neural Sciences, University of Modena and Reggio Emilia, 41125 Modena, Italy; martignazzo@hotmail.it (M.G.); valentina.baldini@unimore.it (V.B.); giuliapisan@gmail.com (G.P.); 2 Department of Biomedical and Neuromotor Sciences, University of Bologna, 40126 Bologna, Italy; 3 Clinic of Child and Adolescent Neuropsychiatry, Department of Mental Health, Physical and Preventive Medicine, University of Campania “Luigi Vanvitelli”, 80138 Naples, Italy; 4 Unit of Dietetics and Sports Medicine, Section of Human Physiology, Department of Experimental Medicine, University of Campania “Luigi Vanvitelli”, 80138 Naples, Italy; giovanni.messina@unicampania.it; 5 Department of Education and Sport Sciences, Pegaso Telematic University, 80143 Naples, Italy; fiorenzo.moscatelli@unipegaso.it (F.M.); maria.ruberto@unipegaso.it (M.R.)

**Keywords:** physical activity, absence seizures, anxiety and depression, quality of life, children

## Abstract

Background: Physical activity is essential for the physical and psychological development of children, contributing to both fitness and overall well-being. However, children with neurological conditions such as childhood absence seizures (CAE), a type of epilepsy characterized by brief episodes of impaired consciousness, may face barriers to participating in regular physical activities. This limitation can negatively affect their quality of life, motor coordination, and cognitive function. Despite this, there is limited research focusing on the physical activity levels of children with absence seizures in comparison to healthy children. Methods: This study aims to compare physical activity levels in children with absence seizures and healthy controls, using the Physical Activity Questionnaire for Children (PAQ-C), a validated tool for assessing children’s engagement in physical activity. The sample included 125 children with absence seizures and 125 healthy controls. The study also assessed anxious–depressive traits using the Children’s Depression Inventory (CDI-2) and the Multidimensional Anxiety Scale for Children (MASC-2). Additionally, seizure frequency and severity were documented for the seizure group, and the impact of different treatment regimens (levetiracetam, valproate, lamotrigine) was explored. Results: The results revealed that children with absence seizures exhibited lower physical activity levels compared to healthy children, although the difference did not reach statistical significance. Furthermore, they had higher scores for anxious–depressive traits. There were no significant differences in physical activity levels between the different treatment groups. The study also found that lower physical activity was correlated with poorer quality of life and increased psychological distress in the seizure group. Conclusion: Children with absence seizures face significant barriers to physical activity, which may be further compounded by psychological distress. These findings emphasize the need for targeted interventions to improve physical activity and address mental health concerns in this population. By enhancing physical activity levels and supporting psychological well-being, interventions can improve the quality of life and overall health of children with absence seizures. Additionally, the results highlight the importance of promoting inclusive physical activity programs for children with neurological conditions.

## 1. Introduction

Childhood Absence Epilepsy (CAE) is a generalized non-convulsive epilepsy characterized by brief, sudden lapses in consciousness, often accompanied by subtle motor symptoms such as eyelid fluttering or slight postural changes, with a rapid return to baseline [1,2]. Although these seizures may appear benign, growing evidence suggests that CAE is frequently associated with cognitive and motor impairments that can persist beyond the ictal episodes [3,4,5]. These deficits often include reduced attention span, executive dysfunction, slowed processing speed, and impaired motor coordination, which can significantly affect academic performance, daily functioning, and participation in physical activity [6,7]. In particular, difficulties in sustained attention and psychomotor speed may interfere with the ability to engage in structured or spontaneous physical play, while fine and gross motor coordination deficits may lead to reduced motor competence and self-efficacy in physical environments.

These impairments, combined with the risk of falls during seizures and the social stigma or overprotection often associated with epilepsy, may contribute to lower levels of physical activity in children with CAE. This is concerning, as physical inactivity during childhood is linked to numerous adverse outcomes, including increased risk of cardiovascular disease, obesity, and poor mental health later in life [8,9,10]. The situation is further compounded by the higher prevalence of anxiety and depression observed in children with absence epilepsy [11,12,13], which may both result from and contribute to physical inactivity through complex bidirectional pathways.

This study investigated the impact of absence seizures on children’s physical activity levels. We examined the influence of anxious–depressive traits and explored whether the type of anti-seizure medication (ASM)—including levetiracetam, valproate, or lamotrigine—modulated physical activity engagement. We hypothesized that children with absence epilepsy would exhibit lower physical activity levels than their healthy peers and that these levels would be inversely associated with anxiety and depression symptoms.

## 2. Methods

### 2.1. Ethical Statement

Written informed consent was obtained from all parents, legal guardians, or caregivers, and verbal assent was secured from the children. The study adhered to the principles outlined in the Declaration of Helsinki and received approval from the Ethics Committee of the University of Campania “Luigi Vanvitelli” (protocol no. 0015300/I, dated 25 June 2020).

### 2.2. Study Design and Setting

This was a cross-sectional, case-control study conducted in the Clinical Unit of Child Neuropsychiatry at Vanvitelli University. The study aimed to compare physical activity levels between children diagnosed with absence epilepsy and healthy controls. Both groups were recruited from the same institution to ensure similarity in environmental and socio-economic factors. Participants were matched for sex and age to minimize demographic biases. Assessment of physical activity was conducted in a blinded manner to reduce potential evaluator bias.

### 2.3. Participants

The study involved 250 children aged between 6 and 12 years. The first group (n = 125) consisted of children diagnosed with absence epilepsy, recruited from the Clinical Unit of Child Neuropsychiatry. The control group (n = 125) included healthy children recruited from the same center, matched for sex and age with the epilepsy group. Inclusion criteria for the seizure group required a clinical diagnosis of absence epilepsy confirmed by an expert child neurologist (MC), while healthy controls were required to have no neurological or psychiatric disorders.

### 2.4. Data Collection

Anthropometric data, including age and sex, were collected for all participants during the initial clinical assessment. However, body weight, height, and body mass index (BMI) were not recorded, as these measures were not part of the standardized assessment protocol and were deemed unrelated to the primary objectives of the study.

Several data points were collected from all participants to ensure a comprehensive assessment of both physical activity and psychological traits. Physical activity levels were assessed using the Physical Activity Questionnaire for Children (PAQ-C), a validated tool that measures children’s activity levels over the past week. To assess anxious–depressive traits, the Children’s Depression Inventory (CDI-2) and the Multidimensional Anxiety Scale for Children (MASC-2) were administered as self-report questionnaires, allowing children to evaluate their own emotional states.

Cognitive function was evaluated using the Italian version of the Wechsler Intelligence Scale for Children-IV (WISC-IV) to control for general cognitive ability. For children with absence seizures, details on the type of ASMs being used, such as levetiracetam, valproate, or lamotrigine, were recorded. Additionally, seizure frequency and severity in the month preceding the study were documented.

**Physical Activity Questionnaire for Children (PAQ-C)** [14]**:**

The PAQ-C is a self-administered, 7-day recall questionnaire designed to assess general levels of physical activity in children aged 8–14 years. It consists of 9 items covering various settings (school, leisure, weekends), each scored from 1 (low activity) to 5 (high activity). The final score is the mean of all items. The PAQ-C has demonstrated good internal consistency (Cronbach’s α = 0.79) and test–retest reliability.

**Children’s Depression Inventory—2 (CDI-2)** [15]**:**

The CDI-2 is a widely used self-report measure for assessing depressive symptoms in children and adolescents aged 7–17. It contains 28 items, each with three response options indicating symptom severity. Total scores can be converted into T-scores, with higher scores reflecting more severe depressive symptoms. The CDI-2 has strong internal consistency (α > 0.85) and construct validity.

**Multidimensional Anxiety Scale for Children—2 (MASC-2)** [16]**:**

The MASC-2 is a standardized instrument used to evaluate anxiety symptoms in children aged 8–19. It comprises 50 items assessing physical symptoms, social anxiety, harm avoidance, and separation anxiety, rated on a 4-point Likert scale. It has excellent psychometric properties, with Cronbach’s α > 0.90 for the total score and strong validity across populations.

**Wechsler Intelligence Scale for Children—Fourth Edition (WISC-IV)** [17]**:**

The WISC-IV is a standardized measure of cognitive functioning in children aged 6–16 years. It provides a Full-Scale IQ based on four composite scores: Verbal Comprehension, Perceptual Reasoning, Working Memory, and Processing Speed. The WISC-IV is extensively validated, with high reliability (α > 0.90) and broad normative data.

### 2.5. Procedures

Each participant underwent a series of assessments. The PAQ-C was administered to both groups to assess physical activity, while the CDI-2 and MASC-2 were used to gauge levels of anxiety and depression in both the seizure and control groups. Children diagnosed with absence epilepsy underwent further clinical evaluations, including EEG recording of seizure characteristics and information on the ASM regimen. Cognitive function was evaluated using the WISC-IV to control for potential confounding factors related to general cognitive ability. All data collection was completed within a designated period, ensuring consistency in the administration of questionnaires and clinical assessments.

## 3. Statistical Analysis

To compare physical activity levels between the two groups, independent samples *t*-tests were used to examine differences in PAQ-C scores. Analysis of Covariance (ANCOVA) was applied to adjust for potential confounding variables such as age and sex. Correlations between PAQ-C scores and anxious–depressive traits (assessed using the CDI-2 and MASC-2) were evaluated using Pearson’s correlation coefficient. A one-way ANOVA was employed to compare PAQ-C scores across the different treatment groups within the seizure group.

For between-group comparisons, a one-way ANOVA was conducted with the F-value reported. Similarly, ANCOVA analyses included the reporting of F-values.

Statistical significance was set at a *p*-value of 0.05. A post hoc power analysis was conducted using a moderate effect size (Cohen’s d = 0.40), an alpha level of 0.05, and a sample size of 125 per group. The resulting power was 0.88, suggesting that the study was sufficiently powered to detect moderate group differences in physical activity levels.

## 4. Results

The study included 250 participants aged 6–12 years: 125 children with absence epilepsy and 125 healthy controls.

The anthropometric characteristics of the participants are summarized in Table 1. No significant differences emerged between the groups in terms of age or sex distribution: in the absence epilepsy (CAE) group, the mean age was 10.26 ± 1.35 years, with 66 females (52.8%) and 59 males (47.2%); in the control group, the mean age was 8.85 ± 1.00 years, with 71 females (56.8%) and 54 males (43.2%).

**Figure 1:**
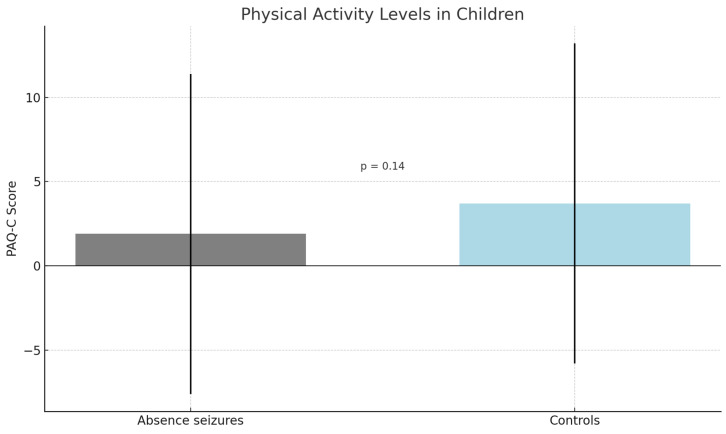
Legend: Mean PAQ-C scores in children with absence seizures and healthy controls. Error bars represent standard deviations. The difference was not statistically significant (*p* = 0.14).

The results indicated a difference, although not statistically significant, in PAQ-C scores between the absence seizure group and the controls (*p* = 0.14). In fact, children with absence seizures reported lower levels of physical activity compared to healthy children (1.9 vs. 3.7) (Figure 1). However, this difference was not statistically significant (*p* = 0.14), likely due to the high within-group variability (SD ≈ 9.5). Children in the absence seizure group had significantly higher scores on both the CDI-2 (*p* < 0.04) and WISC (*p* < 0.001). Children with absence seizures reported higher scores in MASC-2 (63.1 vs. 56.8), indicating higher levels of depressive and anxious symptoms.

A one-way ANOVA comparing PAQ-C scores among children treated with levetiracetam, valproate, and lamotrigine revealed no statistically significant differences (F(2,122) = 1.45, *p* = 0.24) (Table 2).

An ANCOVA was conducted to compare PAQ-C scores between children with absence seizures and controls, adjusting for age and sex. The analysis revealed no statistically significant difference between groups, F(1,246) = 2.18, *p* = 0.14. The effect size was small (partial η^2^ = 0.009). The estimated mean difference was 1.8 points, with a 95% confidence interval ranging from −0.56 to 2.13.

To further explore the potential confounding effect of cognitive functioning, an additional analysis of covariance (ANCOVA) was performed, including WISC-IV full-scale IQ as a covariate, alongside age and sex. The results showed that diagnosis remained a statistically significant predictor of PAQ-C scores (F(1,245) = 128.52, *p* < 0.001), while WISC-IV did not show a significant effect (F(1,245) = 1.94, *p* = 0.165). These results suggest that differences in cognitive functioning do not explain the observed group difference in physical activity levels.

## 5. Discussion

Our results indicate a trend toward lower physical activity levels in children with absence epilepsy compared to the control group, although this difference did not reach statistical significance. The mean physical activity scores for the absence epilepsy group were lower than those for the control group, a finding that aligns with the existing literature and reflects the challenges faced by this population in maintaining typical physical activity routines. Several factors may account for the non-significant result, including a limited sample size and the wide inter-individual variability in children’s activity patterns. Additionally, reliance on a self-reported instrument (PAQ-C) may have introduced response bias and further limited the precision of physical activity assessment, particularly given the cognitive and attentional difficulties often present in pediatric neurodevelopmental populations.

Notably, the significant positive correlations between these emotional health measures and physical activity scores suggest that anxiety and depression may meaningfully influence physical activity engagement. However, it is equally important to consider the potential for reverse causation: that lower levels of physical activity may themselves contribute to the onset or worsening of depressive symptoms. This bidirectional relationship between physical and mental health has been well documented in pediatric research, but causality remains difficult to establish in cross-sectional designs [5,11,12,13,14].

Furthermore, our analysis did not identify a significant effect of anti-epileptic drug (AED) type on physical activity levels. While this result may initially suggest that AED choice does not influence physical activity, it is important to consider potential indirect effects. AEDs may impact physical activity through mechanisms such as cognitive slowing, mood alterations, or motor impairments, which can vary depending on both the medication and the individual’s sensitivity to its side effects. These nuances warrant more detailed investigation in future research.

We acknowledge several limitations in our study. Matching was conducted based on sex and age, given their established relevance and the availability of reliable data. However, key potential confounders—such as body mass index (BMI), socioeconomic status (SES), and parental education level—were not included due to inconsistent data availability across participants. This limits our ability to fully account for the influence of these variables on both physical activity and mental health outcomes. Additionally, the relatively small sample size of the treatment subgroup increases the risk of Type II errors, particularly in subgroup comparisons.

Although the questionnaires employed have demonstrated good test–retest reliability in previous studies, this psychometric property was not assessed in our sample. This limitation is acknowledged and is primarily due to the cross-sectional nature of the study design, which precluded repeated assessments in this pediatric population.

We also recognize that cognitive function may act as an important confounder in the relationship between epilepsy, mental health, and physical activity. This aspect has been addressed in the discussion to provide a more comprehensive understanding of the interrelated factors influencing our findings.

## 6. Conclusions

This study suggests a trend toward lower levels of physical activity in children with absence seizures compared to controls, although this difference did not reach statistical significance. These non-significant differences are nonetheless consistent with prior observations regarding the challenges faced by children with absence epilepsy in maintaining age-appropriate levels of physical activity. The elevated levels of anxiety and depression observed in this group further highlight the potential psychological burden associated with the condition and its possible interplay with physical inactivity.

While the type of anti-epileptic drug (AED) prescribed did not show a statistically significant effect on physical activity levels, further investigation is warranted to explore the influence of other variables—such as seizure frequency and duration, cognitive functioning, and psychosocial support—which may play a more substantial role in determining physical engagement.

Multidisciplinary approaches that address both physical and mental health are recommended to support this vulnerable population. These findings underscore the importance of developing inclusive physical activity programs tailored to the needs of children with neurological conditions and of implementing strategies that enhance their engagement and adherence to such programs.

## Figures and Tables

**Table 1:** Demographic, cognitive, emotional, and physical activity characteristics of children with Childhood Absence Epilepsy (CAE) and matched controls.

Variable	Childhood Absence Epilepsy (CAE) (N = 125)	Controls (N = 125)	*p*-Value
Sex—Female, n (%)	66 (52.8)	71 (56.8)	0.52
Age—M (SD)	10 (1.3)	8.8 (0.9)	0.13
WISC—tot, M (SD)	77.8 (13.5)	108.9 (13.5)	**<0.001**
CDI-2, M (SD)	65 (2.8)	64.8 (2.9)	**0.04**
MASC-2, M (SD)	63.1 (8.3)	56.8 (7.6)	0.13
PAQ-C, M (SD)	1.9 (9.5)	3.7 (9.5)	0.14

Legend: CAE: childhood absence epilepsy; N: number; M: mean; SD: standard deviation; WISC: Wechsler Intelligence Scale for Children; CDI-2: Children’s Depression Inventory, 2nd Edition; MASC-2: Multidimensional Anxiety Scale for Children, 2nd Edition; PAQ-C: Physical Activity Questionnaire for Children. The bold for *p*-value is considered significant when *p* < 0.05 (statistically significant). Independent samples *t*-tests were used for all continuous variables; the Chi-square test was used for sex.

**Table 2:** Legend: PAQ-C: Physical Activity Questionnaire for Children.

Variable	Levetiracetam	Valproate	Lamotrigine	*p* Value
PAQ-C, M	1.9	1.8	1.8	0.24

## Data Availability

The original contributions presented in the study are included in the article, further inquiries can be directed to the corresponding author.
